# International Survey to Establish Prioritized Outcomes for Trials in People With Coronavirus Disease 2019

**DOI:** 10.1097/CCM.0000000000004584

**Published:** 2020-08-18

**Authors:** Nicole Evangelidis, Allison Tong, Martin Howell, Armando Teixeira-Pinto, Julian H. Elliott, Luciano Cesar Azevedo, Andrew Bersten, Lilia Cervantes, Derek P. Chew, Sally Crowe, Ivor S. Douglas, Ella Flemyng, Peter Horby, Jaehee Lee, Eduardo Lorca, Deena Lynch, John C. Marshall, Anne McKenzie, Sangeeta Mehta, Mervyn Mer, Andrew Conway Morris, Saad Nseir, Pedro Povoa, Mark Reid, Yasser Sakr, Ning Shen, Alan R. Smyth, Tom Snelling, Giovanni F. M. Strippoli, Antoni Torres, Tari Turner, Steve Webb, Paula R. Williamson, Laila Woc-Colburn, Junhua Zhang, Amanda Baumgart, Sebastian Cabrera, Yeoungjee Cho, Tess Cooper, Chandana Guha, Emma Liu, Andrea Matus Gonzalez, Charlie McLeod, Patrizia Natale, Valeria Saglimbene, Andrea K. Viecelli, Jonathan C. Craig

**Affiliations:** 1 Sydney School of Public Health, The University of Sydney, Sydney, NSW, Australia.; 2 Centre for Kidney Research, The Children's Hospital at Westmead, NSW, Australia.; 3 Cochrane Australia, School of Public Health and Preventive Medicine, Monash University, Melbourne, VIC, Australia.; 4 Department of Critical Care Medicine, Hospital Sirio-Libanes, São Paulo, Brazil.; 5 College of Medicine and Public Health, Flinders University, Adelaide, SA, Australia.; 6 Department of Medicine, Denver Health, Denver, CO.; 7 Crowe Associates Ltd, Oxon, United Kingdom.; 8 Department of Medicine, Pulmonary Sciences and Critical Care, Denver Health and University of Colorado Anschutz, School of Medicine, Denver, CO.; 9 Editorial and Methods Department, Cochrane, London, United Kingdom.; 10 Nuffield Department of Medicine, University of Oxford, Oxford, United Kingdom.; 11 Department of Internal Medicine, School of Medicine, Kyungpook National University, Daegu, South Korea.; 12 Department of Internal Medicine, Faculty of Medicine, University of Chile, Santiago, Chile.; 13 Jonze Society, Brisbane, Australia.; 14 Department of Surgery, University of Toronto, Toronto, ON, Canada.; 15 Telethon Kids Institute, Perth, WA, Australia.; 16 Department of Medicine and Interdepartmental Division of Critical Care Medicine, University of Toronto, Toronto, ON, Canada.; 17 Divisions of Critical Care and Pulmonology, Department of Medicine, Charlotte Maxeke Johannesburg Academic Hospital and Faculty of Health Sciences, University of the Witwatersrand, Johannesburg, South Africa.; 18 Department of Medicine, University of Cambridge, Cambridge, United Kingdom.; 19 Critical Care Centre, CHU Lille, and Lille University, F-59000 Lille, France.; 20 Nova Medical School, CHRC, New University of Lisbon, Polyvalent Intensive Care Unit, Sao Francisco Xavier Hospital, CHLO, Lisbon, Portugal. Center for Clinical Epidemiology and Research Unit of Clinical Epidemiology, OUH Odense University Hospital, Denmark.; 21 Department of Anesthesiology and Intensive Care, Jena University Hospital, Jena, Germany.; 22 Department of Respiratory Medicine, Peking University Third Hospital, Beijing, China.; 23 Evidence Based Child Health Group, University of Nottingham, Nottingham, United Kingdom.; 24 Department of Emergency and Organ Transplantation, University of Bari, Bari, Italy.; 25 Department of Pulmonology Hospital Clinic. University of Barcelona, CIBERES, IDIBAPS, ICREA, Barcelona, Spain.; 26 Department of Biostatistics, University of Liverpool, Liverpool, United Kingdom.; 27 Section of Infectious Diseases Department of Medicine, National School of Tropical Medicine Baylor College of Medicine, Houston, TX.; 28 Evidence-based Medicine center, Tianjin University of Traditional Chinese Medicine, Tianjin, China.; 29 Faculty of Medicine, University of Queensland, Brisbane, Old, Australia.; 30 Department of Infectious Diseases, Perth Children’s Hospital, Perth, WA, Australia.

**Keywords:** clinical trial, coronavirus, critical care, infection, patients, sepsis

## Abstract

**Objectives:**

There are over 4,000 trials conducted in people with coronavirus disease 2019. However, the variability of outcomes and the omission of patient-centered outcomes may diminish the impact of these trials on decision-making. The aim of this study was to generate a consensus-based, prioritized list of outcomes for coronavirus disease 2019 trials.

**Design:**

In an online survey conducted in English, Chinese, Italian, Portuguese, and Spanish languages, adults with coronavirus disease 2019, their family members, health professionals, and the general public rated the importance of outcomes using a 9-point Likert scale (7–9, critical importance) and completed a Best-Worst Scale to estimate relative importance. Participant comments were analyzed thematically.

**Setting:**

International.

**Subjects:**

Adults 18 years old and over with confirmed or suspected coronavirus disease 2019, their family members, members of the general public, and health professionals (including clinicians, policy makers, regulators, funders, and researchers).

**Interventions:**

None.

**Measurements:**

None.

**Main Results:**

In total, 9,289 participants from 111 countries (776 people with coronavirus disease 2019 or family members, 4,882 health professionals, and 3,631 members of the public) completed the survey. The four outcomes of highest priority for all three groups were: mortality, respiratory failure, pneumonia, and organ failure. Lung function, lung scarring, sepsis, shortness of breath, and oxygen level in the blood were common to the top 10 outcomes across all three groups (mean > 7.5, median ≥ 8, and > 70% of respondents rated the outcome as critically important). Patients/family members rated fatigue, anxiety, chest pain, muscle pain, gastrointestinal problems, and cardiovascular disease higher than health professionals. Four themes underpinned prioritization: fear of life-threatening, debilitating, and permanent consequences; addressing knowledge gaps; enabling preparedness and planning; and tolerable or infrequent outcomes.

**Conclusions:**

Life-threatening respiratory and other organ outcomes were consistently highly prioritized by all stakeholder groups. Patients/family members gave higher priority to many patient-reported outcomes compared with health professionals.

## Abstract 2

Supplemental Digital Content is available in the text.

As of July 8, 2020, over 11.6 million cases of coronavirus disease 2019 (COVID-19) have been reported, with more than 539,000 deaths in 216 countries (1). The risk of death in patients hospitalized for COVID-19 is estimated to range from 12% to 33% (2–7). In a study of 5,700 hospitalized patients in the United States, 14% required ICU admission, and 12% received invasive mechanical ventilation (8). Patients with COVID-19 experience fatigue, cough, and dyspnea, which impair daily functioning (4, 9–11), and mental health sequelae such as depression and anxiety are of concern (12–14).

Substantial resources have been invested in clinical trials in COVID-19 (15), with more than 4,000 trials registered on the World Health Organization (WHO) International Clinical Trials Registry Platform. However, the outcomes included are inconsistent, and patient-reported outcomes (16) are infrequently measured. Serious clinical outcomes (e.g., mortality, respiratory failure) are not always reported, and patient-reported outcomes (e.g., cough) are rarely included (17–19). These problems can diminish the relevance of the evidence for decision-making by patients, health professionals, and regulators.

Three core outcome sets for COVID-19 have been developed, with respiratory failure and mortality in hospitalized patients common to all (20–22). However, only one of these initiatives involved consultation with patients and the public (*n* = 27), all of whom were from China. The global COVID-19- Core Outcomes Set (COS) initiative convened in March 2020, to bring together people with COVID-19 and family members, members of the general public, and health professionals, to establish consensus-based core outcomes for trials in people with confirmed or suspected COVID-19 across the full spectrum of disease, in a two-step process. First this study, and then an online series of workshops. The aim of this study was to identify a prioritized list of outcomes for trials in COVID-19 and to explain the reasons behind the prioritization decisions.

## MATERIALS AND METHODS

We used the Core Outcome Set Standards for Reporting Statement (23).

### Study Design

We followed the Core Outcome Measures in Effectiveness Trials (COMET) methodological framework, which involves a Delphi survey and a consensus workshop (24). The consensus workshops will be published separately. This international survey was initially designed to be a two-round Delphi survey to generate consensus (24). Consensus was achieved in round 1, making round 2 unnecessary. Participants prioritized outcomes for trials in people with confirmed or suspected COVID-19. The survey was conducted in English, Chinese, Italian, Spanish, and Portuguese.

### Participant Selection and Recruitment

People 18 years old and over with confirmed or suspected COVID-19 and their family members, members of the public, and health professionals (including clinicians, researchers, and policy makers) were eligible. We used multiple recruitment strategies to be as broadly inclusive and diverse as possible. Respondents were recruited through the Steering Committee and investigator networks and professional and consumer organizations using email, social media, and a market research company. The University of Sydney provided ethics approval.

### Data Collection

#### Selection of Outcome Domains.

We included outcome domains from systematic reviews and published core outcome sets for COVID-19, after discussion among the Steering Committee, which included patients and members of the public (20–22, 25) (**eTable 1**, Supplemental Digital Content 1, http://links.lww.com/CCM/F730) The order of outcomes was randomized. The survey was administered online using Qualtrics software (SAP, Provo, UT). The survey was open for 16 days from March 31, 2020, to April 16, 2020.

#### Prioritization of Outcomes.

Participants rated the importance of each of the 25 outcome domains using the Grading of Recommendations Assessment, Development, and Evaluation process 9-point Likert scale (26). Scores 1–3 indicated “limited importance,” 4–6 indicated “important but not critical,” and 7–9 indicated “critical importance.” Participants could enter comments in free-text boxes and suggest new outcomes. Participants also completed a Best-Worst Scale survey comprising of five-choice sets, including six of the possible 25 outcomes. The Best-Worst Scale survey is a well-established method for eliciting relative preferences. The outcomes included in each set were determined using a balanced, incomplete block design (27, 28). Participants selected the most important and least important outcome from each block of outcomes presented.

### Data Analysis

#### Quantitative Analysis.

We calculated the mean score, median, and proportion of participants who rated the outcome from 7 to 9 (critically important) for each outcome. We calculated the scores separately for patients/family, members of the public, and health professionals. We compared the “public” and “health professional” group with the “patients/family” group using mean differences. We did not rely on the normality assumption and used nonparametric tests to compare the importance of the outcome given by the three groups. The overall differences were compared using Kruskal-Wallis tests. If significant differences were found, we performed the pairwise comparisons using Mann-Whitney *U* tests with Bonferroni correction to adjust for multiple comparisons. The relative importance score was derived using a multinomial logistic regression model. Utility functions containing all outcomes and interaction terms for participant characteristics were constructed for the Best-Worst Survey. Following this approach, the mean regression coefficients of this function provided the relative importance scores for each outcome (27). The regression coefficients have the same underlying scale; therefore, for ease of interpretation, we used a scale of 1 (least important) to 9 (most important). Statistical analyses were conducted using SPSS (Version 25.0; IBM SPSS Statistics for Windows, Armonk, NY) and NLOGIT 6 (Econometric Software, Plainview, NY). A *p* value of less than 0.05 was considered statistically significant.

#### Definition of Consensus.

The thresholds for consensus could not be defined a priori because the distribution of scores was not known prior to data collection. After reviewing the results, we set the threshold to identify the top 10 outcome domains indicated as critically important by all three stakeholder groups on the Likert scale. This was based on the patient/family, public, and health professional groups, each having mean greater than 7.5, median greater than or equal to 8, and greater than 70% of each group rating the outcome as “critically important.” The 10 outcomes of highest priority were used to select the proposed 3 to 5 core outcomes to discuss at the consensus workshop.

#### Qualitative Analysis.

Survey comments were imported into Hyper RESEARCH (ResearchWare Inc, version 3.7; Randolph, MA) software for data analysis. Investigators (N.E., A.T.) used thematic analysis to code the text and inductively identify themes to explain the reasons for prioritizing outcomes among stakeholder groups.

## RESULTS

### Participant Characteristics

In total, 9,289 participants from 111 countries completed the survey, of whom 776 (8%) were patients with confirmed or suspected COVID-19 or their family members, 4,882 (53%) were health professionals, and 3,631 (39%) were members of the public. The participant characteristics are provided in **Table 1**. The 776 patients/family members were from 40 countries. Seventy-one of the 590 patients (12%) with suspected or confirmed COVID-19 reported having pneumonia, 21 (4%) reported organ failure, and 20 (3%) sepsis. Fifty (8%) were treated with oxygen and 13 (2%) required mechanical ventilation. Of the 4,882 health professionals (from 95 countries), 2,249 (46%) were physicians, 1,218 were researchers (25%), 866 (18%) were nurses, 78 (2%) were psychologists, 55 (1%) were policymakers, 48 (1%) were social workers, 37 (1%) were industry representatives, and 1,153 (24%) were “other health professionals.” Among the physicians, 364 (16%) specialized in internal medicine, 320 (14%) were intensive/critical care specialists, 155 (7%) were general practitioners, 103 (5%) were emergency physicians, 101(4%) were infectious disease specialists, 98 (4%) were pulmonary/respiratory specialists, and 97 (4%) were public health physicians, and 1,011 (45%) were “other specialist physicians.” The 3,631 members of the general public were from 76 countries.

**TABLE 1.** Participant Characteristics (*n* = 9,289)

Characteristic	Patients/Family Members, *n* = 776, *n* (%)	Health Professionals, *n* = 4,882, *n* (%)	Public, *n* = 3,631, *n* (%)
Participant type^a^			
Person with suspected COVID-19	420 (54)	—	—
Person with confirmed COVID-19	170 (22)	—	—
Family/caregiver	312 (40)	—	—
Clinician	215 (28)	3,295 (67)	—
Researcher	123 (16)	1,480 (30)	—
Policy maker	23 (3)	69 (1)	—
Other health professional	41 (5)	813 (17)	—
Gender^b^			
Male	253 (33)	1,578 (32)	1,221 (34)
Female	510 (66)	3,265 (67)	2,381 (66)
Age group (yr)			
18–29	131 (17)	1,190 (24)	763 (21)
30–39	203 (26)	1,441 (30)	629 (17)
40–49	213 (27)	1,036 (21)	755 (21)
50–59	133 (17)	772 (16)	590 (16)
60–69	74 (10)	370 (8)	599 (16)
70 and over	22 (3)	73 (1)	295 (8)
Currently in self-isolation/quarantine			
Yes	514 (66)	904 (19)	1,479 (41)
No	262 (34)	3,978 (81)	2,152 (59)
Language of survey completion			
English	584 (75)	3,004 (62)	2,339 (64)
Chinese	49 (6)	1,075 (22)	356 (10)
Italian	59 (8)	253 (5)	362 (10)
Spanish	47 (6)	298 (6)	307 (8)
Portuguese	37 (5)	252 (5)	267 (7)
Preexisting/health conditions^a^			
High blood pressure/cardiovascular/heart conditions	150 (19)	—	718 (20)
Respiratory condition (e.g., asthma)	123 (16)	—	382 (11)
Diabetes/obesity	120 (15)	—	477 (13)
Other/s	169 (22)		964 (27)
None	408 (53)	—	1,972 (54)
Symptoms^a^ (suspected or confirmed patients only, *n* = 590)			
Fatigue	393 (67)	—	—
Cough	361 (61)	—	—
Fever	312 (53)	—	—
Shortness of breath	243 (41)	—	—
Loss of taste and/or smell	228 (39)	—	—
Nausea, vomiting, diarrhea	202 (34)	—	—
Chest pain	201 (34)	—	—
Other	223 (38)	—	—
None/do not know	67 (11)	—	—
Complications^a^ (suspected or confirmed patients only, *n* = 590)			
Pneumonia	71 (12)	—	—
Organ failure	21 (4)	—	—
Sepsis	20 (3)	—	—
None	451 (76)	—	—
Other	58 (10)	—	—
Treatment^a^ (suspected or confirmed patients only, *n* = 590)			
Antiviral therapy	50 (8)	—	—
Oxygen	50 (8)	—	—
Hydroxychloroquine/chloroquine	41 (7)	—	—
Mechanical ventilation	13 (2)	—	—
Other/s	115 (19)	—	—
None/do not know	394 (67)	—	—
Country			
United Kingdom	268 (35)	753 (15)	772 (21)
China	51 (7)	1,113 (23)	370 (10)
United States	146 (19)	461 (9)	787 (22)
Australia	50 (6)	765 (16)	390 (11)
Italy	58 (7)	280 (6)	355 (10)
Other^c^	206 (27)	1,532 (31)	975 (27)

COVID-19 = coronavirus disease 2019.

^a^Percentages may not add up to 100 due to multiple selections.

^b^Prefer not to say: patients /family members 13 (2%), health professionals 39 (1%), and public 29 (1%).

^c^Other includes 106 countries in descending order of total number of participants: Portugal, Canada, Brazil, Mexico, Spain, Chile, France, Netherlands, Ireland, Germany, New Zealand, India, Switzerland, South Africa, Argentina, Belgium, Iceland, Sweden, Nigeria, Colombia, Costa Rica, Singapore, United Arab Emirates, Japan, Guatemala, Angola, Korea, Republic of, Saudi Arabia, Lithuania, Uganda, Norway, Denmark, Pakistan, Greece, Poland, Uruguay, Austria, Dominican Republic, El Salvador, Malaysia, Turkey, Cameroon, Kenya, Peru, Panama, Egypt, Bangladesh, Ecuador, Islamic Republic of Iran, Iraq, Israel, Gambia, Indonesia, Malawi, Philippines, Serbia, Zimbabwe, Albania, Bulgaria, Ethiopia, Jordan, Kuwait, Malta, Paraguay, Russian Federation, Sri Lanka, Côte d’Ivoire, Estonia, Honduras, Qatar, Senegal, Trinidad and Tobago, Tunisia, Ukraine, United Republic of Tanzania, Bolivarian Republic of Venezuela, Viet Nam, Marshall Islands, Yemen, Afghanistan, Andorra, Antigua and Barbuda, Barbados, Belize, Botswana, Cape Verde, Czech Republic, Finland, Georgia, Hungary, Lebanon, Liechtenstein, Luxembourg, Mozambique, Nepal, Oman, Puerto Rico, Romania, Rwanda, Sao Tome and Principe, Slovakia, Slovenia, Sudan, Thailand, Timor-Leste, and Zambia.

Dashes indicate data not applicable.

### Absolute Importance Scores

**Figure 1.**
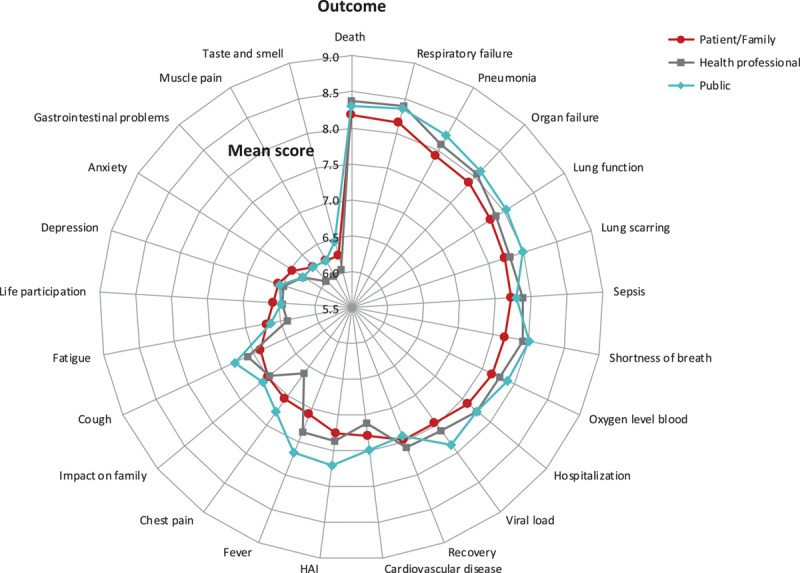
Mean scores of patients/family members, health professionals, and the general public (ordered by patient/family scores). Total sample size differs for each outcome. Death, cardiovascular disease, shortness of breath, chest pain, and cough (*n* = 9,289); fever, pneumonia, hospitalization, depression, and impact on family (*n* = 9,072); gastrointestinal problems, fatigue, life participation, muscle pain, and respiratory failure (*n* = 8,907); sepsis/septic shock, taste and smell, anxiety, lung function, and recovery (*n* = 8,789); and oxygen level in the blood, viral load or clearance, lung scarring (fibrosis), hospital-acquired infection (HAI), and organ failure *n* = 8,653.

The top four outcomes with the highest mean score for patients/family members, health professionals, and the public were the same (**Fig. 1**; **eTables 2** and **3**, Supplemental Digital Content 1, http://links.lww.com/CCM/F730). This included death (patients/family: mean 8.2, health professionals: mean 8.4, and public: mean 8.3); respiratory failure (patients/family: 8.2, health professionals: 8.4, and public: 8.4); pneumonia (patients/family: 7.9, health professionals: 8.1, and public: 8.2); and organ failure (patients/family: 7.9, health professionals: 8.1, and public: 8.1). Lung function, lung scarring, sepsis, shortness of breath, and oxygen level in the blood were in the top ten outcomes for each stakeholder group.

**Figure 2.**
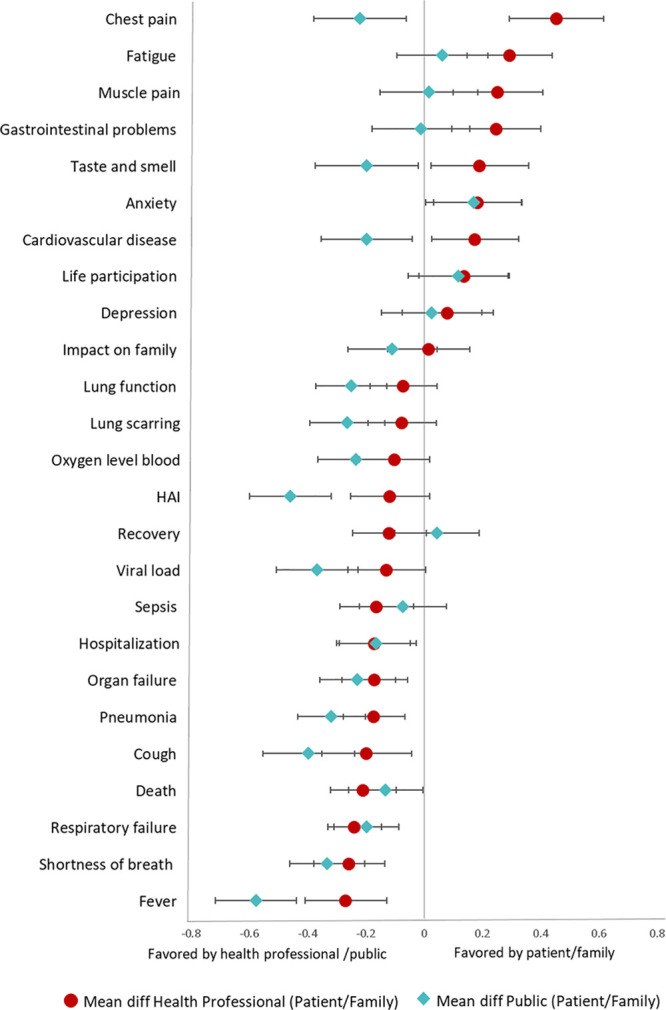
Difference in mean scores among patients/family members, public, and health professionals. Patients/family members are the reference group. The distance between each value represents the difference in mean scores between groups (patients/family members—health professionals; patients/family members—public). For example, patients/family members rated chest pain higher than health professionals (mean score difference of 0.45). Health professionals rated fever higher than patients/family members (mean score difference of –0.27). diff = difference, HAI = hospital-acquired infection.

Differences in mean scores among patients/family, health professionals, and the public are shown in **Figure 2**. Patients/family rated the following six outcomes higher than health professionals on the Likert scale: chest pain (absolute mean difference, 0.5; *p* < 0.001), fatigue (0.3; *p* < 0.001), muscle pain (0.3; *p* = 0.003), gastrointestinal problems (0.3; *p* = 0.005), anxiety (0.2; *p* = 0.034), and cardiovascular disease (0.2; *p* < 0.001). Health professionals rated the following four outcomes higher than patients/family on the Likert scale: fever (0.3; *p* < 0.001), shortness of breath (0.3; *p* < 0.001), respiratory failure (0.2; *p* = 0.002), and cough (0.2; *p* = 0.042).

### Relative Preferences—Best-Worst Scale

**Figure 3.**
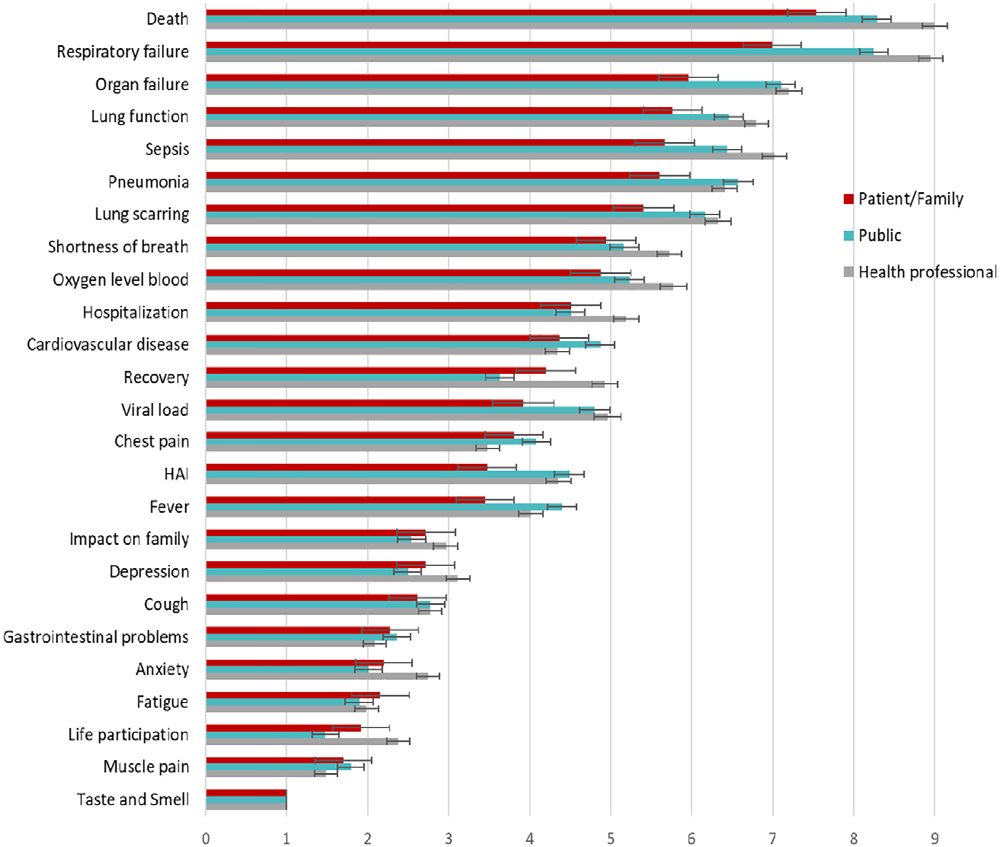
Mean relative importance scores of patients/family members, public, and health professionals based on the Best-Worst Scale, ordered by the mean importance scores of patients/family members (*error bars* are 95% CI). HAI = hospital-acquired infection.

Patients/family members considered death to be the most important, followed by respiratory failure, organ failure, lung function, and sepsis (**Fig. 3**). The differences between death and respiratory failure were small with preference scores of 7.5 (95% CI, 7.2–7.9) and 6.6 (95% CI, 6.6–7.4), respectively. Death and respiratory failure were also ranked as the top two most important outcomes by the public and health professionals. However, they were of equal importance with preference scores for death and respiratory failure being 8.3 (95% CI, 8.1–8.5) and 8.2 (95% CI, 8.1–8.4) for the public and 9.0 (95% CI, 8.8–9.2) and 9.0 (95% CI, 8.8–9.1) for health professionals. Shortness of breath the most important patient-reported outcome for patients/family, the public, and health professionals.

**Figure 4.**
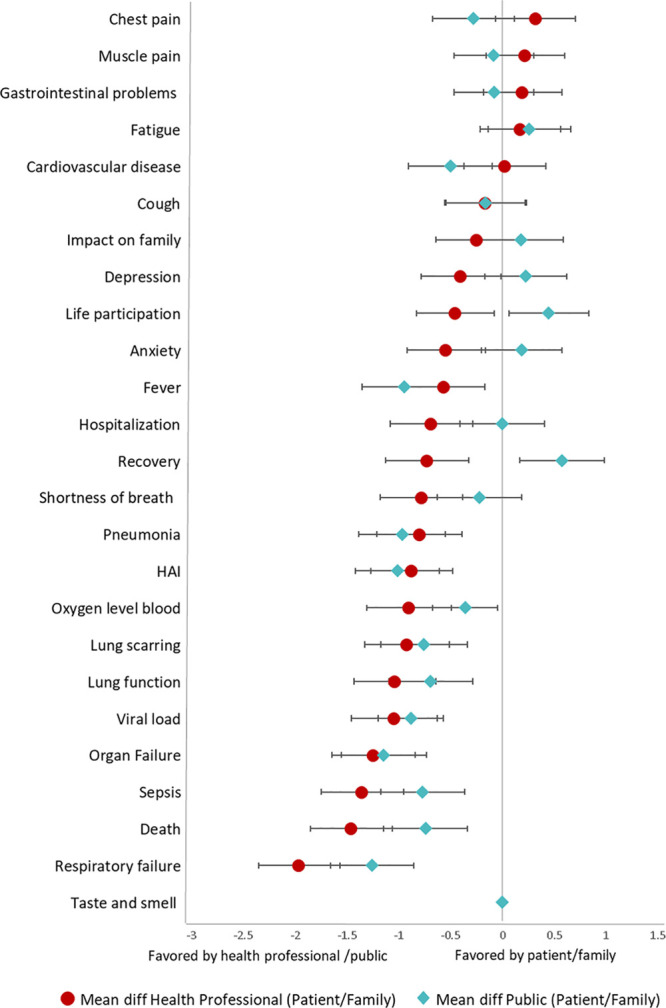
Difference in mean scores among patients/family members, public, and health professionals based on the Best-Worst Scale. Patients/family members are the reference group. diff = difference, HAI = hospital-acquired infection.

The differences in mean preferences scores between patients/family and health professionals and the public were generally small, being less than 2.0 (scale 1 to 9) for all outcomes (**Fig. 4**). Compared to patients/family, the most notable differences were for death (mean differences in relative preference scores of –1.3 [95% CI, –1.7 to –0.9] and –2.0 [95% CI, –2.4 to –1.6] for the public and health professionals, respectively) and respiratory failure (mean differences of –1.1 [95% CI, –1.6 to –0.7] and –1.2 [95% CI, –1.6 to –0.8] for the public and health professionals, respectively). Impact on family, anxiety, depression, fatigue, recovery, and life participation were all ranked as more important by patients/family members compared to the public; chest pain, muscle pain, gastrointestinal outcomes, and fatigue were ranked as more important by patients/family compared to health professionals.

The mean, median, and Best-Worst Scale scores according to survey language (English, Chinese, Italian, Spanish, and Portuguese) are provided in **eTable 4** and **eFigure 1** (Supplemental Digital Content 1, http://links.lww.com/CCM/F730). The 10 most highly prioritized outcomes by country were generally similar. Respiratory failure was in the top two for all languages. Based on the mean score, death was first in the English survey, third in the Portuguese survey, fourth for Chinese, and sixth for the Spanish and Italian surveys. Subgroup analysis by disease severity was not possible due to the limited number of patients with severe disease.

### Additional Outcomes

Thirteen additional outcomes were each suggested by 30 or more respondents, including post-traumatic stress disorder (PTSD), anemia/iron, delirium, diabetes, cognition, immunity and antibodies, dizziness, dysphagia, acute kidney injury, financial impact, headache, and physical function.

### Themes From Comments

We identified four themes underpinning the prioritization of outcomes, which are described below. Selected quotations to support each theme are provided in **eFigure 2** (Supplemental Digital Content 1, http://links.lww.com/CCM/F730).

#### Fear of Life-Threatening, Debilitating, and Permanent Consequences.

Participants gave higher priority to outcomes that were seen as a threat to survival—“(respiratory failure) is thought to be the main cause of death.” The visible risk of death explained the high priority for mortality—“you see death coming for you” and that you “imagine the worse possible scenario, particularly when you are short of breath.” Severe outcomes that required isolation or admission to intensive care or invasive interventions were distressing and “could be linked to longer-term issues such as PTSD.” Symptoms that “lingered” and impaired quality of life (e.g., shortness of breath) were given higher priority. For patients, the high prioritization of impact on family, depression, and anxiety reflected angst about isolation and profound guilt of infecting others. Anxiety was also rated high because it exacerbated symptoms—“this uncertainty causes anxiety and makes breathing harder.”

#### Addressing Knowledge Gaps.

Because of the uncertain trajectory and prognosis of COVID-19, participants gave higher priority to outcomes related to disease progression (e.g., mortality, respiratory failure, pneumonia, lung function, and recovery). Some gave higher priority to outcomes they believed were specific to COVID-19 (e.g., respiratory failure, shortness of breath) over those regarded to be more general (e.g., anxiety) or may have been preexisting.

#### Enabling Preparedness and Planning.

Outcomes were given higher priority if they had implications for healthcare resource use. Respiratory failure was a high priority because it could require admission into intensive care and interventions including oxygen, mechanical ventilation, and extracorporeal membrane oxygenation—“it informs all measures that should be taken (lockdown, production of ventilators, etc.)” Some also considered outcomes for decision-making—“this would inform my decision to continue very invasive treatment or not.”

#### Tolerable or Infrequent Outcomes.

Participants gave lower priority for outcomes such as cough, gastrointestinal symptoms, fever, and muscle pain because these were perceived to be manageable, uncommon, or transient. Some patients based their decisions on whether they had experienced the symptom personally.

## DISCUSSION

For patients, family members, health professionals, and the public, mortality, respiratory failure, pneumonia, organ failure, lung function, lung scarring, sepsis, shortness of breath, and blood oxygen were the outcomes of highest priority. Mortality, respiratory failure, pneumonia, and organ failure of highest priority for all three groups. The patient-reported outcome of highest priority for all groups was shortness of breath. Hospitalization was in the top 10 for patients/family and health professionals. Viral load/clearance was in the top 10 for the public. Recovery was the top-rated long-term outcome. The top prioritized outcomes reflected the prevailing fear of death and debilitating and permanent consequences, uncertainty of disease progression, and concerns about the burden of disease on health systems.

Mortality and respiratory failure have also been identified in the three core outcomes set previously established for COVID-19 (20–22), albeit with previous initiatives focused on hospitalized patients only. The WHO core outcome measures set for COVID-19 include viral burden and a clinical progression scale that includes five states: uninfected, ambulatory, hospitalized with mild disease, hospitalized with severe disease, and death. The “ambulatory” state refers to the patient being symptomatic. However, no specific symptoms were identified. Shortness of breath, recovery (defined as feeling better, no longer having symptoms), and chest pain were the three patient-reported outcomes of highest importance as identified in our survey. Of note, chest pain, fatigue, muscle pain, and gastrointestinal problems were rated higher by patients/family compared with health professionals. Anxiety was rated higher by patients/family compared with both health professionals and the general public. People with COVID-19 attributed anxiety to the fear and guilt of infecting others and the need to be quarantined and isolated from family and support persons. Anxiety exacerbated their symptoms, particularly shortness of breath. The inclusion of patients and the public brings nuanced insights and attention to include outcomes of importance (e.g., shortness of breath) that may be underrecognized and not explicitly included in core outcome sets.

The survey included a large number of participants (*n* = 9,289) from 111 countries, with 47% comprising of patients, family members, and the public. The survey was available in five languages. The data were triangulated by assessing both the absolute and relative importance of scores, using four measures (mean, median, proportion, and Best-Worst Scale score). However, there are some potential limitations. The online survey would have precluded involvement of those who do not have access to the internet. Most participants were from high-income countries and completed the English language survey, and only a small number of patients with severe disease were included. Not all participants completed the entire survey; however, we have indicated the number of responses for each outcome and of those who completed the Likert ratings and Best-Worst Scale.

## CONCLUSIONS

Mortality, respiratory failure, organ failure, sepsis, and shortness of breath are of critical importance to people with confirmed or suspected COVID-19 and their family members, the public, and health professionals. These outcomes will comprise the proposed core outcomes to be reviewed and discussed at international, online multi-stakeholder consensus workshops to establish the core outcome domains for COVID-19. The use of outcomes of importance to all stakeholders, including patients, the public, and health professionals, can help to improve the relevance of trials to better inform decision-making in practice and policy.

## Supplementary Material


