# Supplementary material for: International Survey to Establish Prioritized Outcomes for Trials in People With Coronavirus Disease 2019
Source: Crit Care Med. 2020 Aug 18;48(11):1612–21. doi: 10.1097/CCM.0000000000004584 (PMC7448718; doi:10.1097/CCM.0000000000004584)

**eTable 1. Outcomes and descriptors in the COVID-19-COS survey**

| **No** | **Outcome and definition** |
| --- | --- |
|  |  |
| **1** | **Death**  *Number of people who die, risk of death* |
| **2** | **Cardiovascular disease**  *Disease of the heart and blood vessels e.g. heart attack, stroke* |
| **3** | **Shortness of breath**  *Difficult or discomfort in breathing (dyspnea)* |
| **4** | **Chest pain**  *Pain or pressure in the chest* |
| **5** | **Cough**  *Needing to clear the throat and breathing passage* |
| **6** | **Fever**  *Abnormally high body temperature* |
| **7** | **Pneumonia**  *Infection of the lungs* |
| **8** | **Hospitalization**  *Staying in hospital overnight or longer, including time to discharge, admission/length of stay in the intensive care unit (ICU)* |
| **9** | **Depression**  *Strong feelings of sadness, hopelessness, despair for most of the time, for more than two weeks* |
| **10** | **Impact on family**  *Physical, psychological and emotional impacts on the family* |
| **11** | **Gastrointestinal problems**  *Uneasy feeling in the stomach, wanting to throw up, vomiting, diarrhea* |
| **12** | **Fatigue**  *Feeling tired with no energy for weeks, for most of the time, which can interfere with normal daily life* |
| **13** | **Life participation**  *Being able to do meaningful activities of life (e.g. hobbies, study, work, social activities), includes social isolation* |
| **14** | **Muscle pain**  *Pain in a muscle or group of muscles (myalgia)* |
| **15** | **Respiratory failure**  *When help of either an oxygen mask or possibly a machine is required to provide enough oxygen (respiratory support).* |
| **16** | **Sepsis / septic shock**  *A life-threatening condition that occurs when widespread infection leads to dangerously low blood pressure and organ damage or failure* |
| **17** | **Taste and smell**  *Loss of sense of taste and/or smell (anosmia, ageusia)* |
| **18** | **Anxiety**  *Persistent stress and worry* |
| **19** | **Lung function**  *How well your lungs and airways are working* |
| **20** | **Recovery**  *How long it takes to recover (feeling better, no longer having symptoms)* |
| **21** | **Oxygen level in the blood**  *Level of oxygen in the blood, oxygen saturation, low levels may require oxygen therapy* |
| **22** | **Viral load or clearance**  *Amount of virus in the body* |
| **23** | **Lung scarring (fibrosis)**  *Damage and scarring of the lung tissue, which may lead to long-term breathing difficulties* |
| **24** | **Hospital-acquired infection**  *An infection that occurs as a result of medical treatment* |
| **25** | **Organ failure (excludes respiratory)**  *Failure of one or more organ systems (e.g. cardiovascular, kidney)* |

**eTable 2. Means, medians and proportion (rating 7-9)^†^ of patients/family members, health professionals and public for 25 outcomes.** Ordered by patient/family mean scores.

|  | **Patients/Family n=776** | | | **Health Professional n=4882** | | | **Public n=3631** | | | **Mean differences between groups** | | | |
| --- | --- | --- | --- | --- | --- | --- | --- | --- | --- | --- | --- | --- | --- |
|  | **Mean** | **Median** | **%** | **Mean** | **Median** | **%** | **Mean** | **Median** | **%** | **Overall*** | **Patients/family & Health professional**** | **Patients/family & Public**** | **Health professional & Public**** |
| Death | 8.2 | 9.0 | 81 | 8.4 | 9.0 | 87 | 8.3 | 9.0 | 82 | 0.268 |  |  |  |
| Respiratory failure | 8.2 | 9.0 | 86 | 8.4 | 9.0 | 92 | 8.4 | 9.0 | 89 | <0.001 | 0.002 | <0.001 | 0.298 |
| Pneumonia | 7.9 | 9.0 | 83 | 8.1 | 9.0 | 87 | 8.2 | 9.0 | 87 | <0.001 | 0.202 | <0.001 | <0.001 |
| Organ failure | 7.9 | 9.0 | 81 | 8.1 | 9.0 | 85 | 8.1 | 9.0 | 83 | <0.001 | >0.999 | 0.001 | <0.001 |
| Lung function | 7.8 | 8.0 | 81 | 7.9 | 8.0 | 83 | 8.0 | 9.0 | 84 | <0.001 | >0.999 | <0.001 | <0.001 |
| Lung scarring (fibrosis) | 7.7 | 8.0 | 77 | 7.8 | 8.0 | 81 | 8.0 | 9.0 | 81 | <0.001 | >0.999 | <0.001 | <0.001 |
| Sepsis / septic shock | 7.7 | 9.0 | 77 | 7.9 | 9.0 | 80 | 7.8 | 9.0 | 74 | 0.268 |  |  |  |
| Shortness of breath | 7.7 | 8.0 | 77 | 7.9 | 9.0 | 81 | 8.0 | 9.0 | 81 | <0.001 | <0.001 | <0.001 | 0.003 |
| Oxygen level in the blood | 7.7 | 8.0 | 76 | 7.8 | 8.0 | 80 | 7.9 | 8.0 | 79 | <0.001 | 0.925 | <0.001 | <0.001 |
| Hospitalization | 7.6 | 8.0 | 75 | 7.8 | 8.0 | 80 | 7.7 | 8.0 | 76 | 0.048 | 0.358 | 0.058 | 0.419 |
| Viral load or clearance | 7.5 | 8.0 | 69 | 7.6 | 8.0 | 73 | 7.8 | 8.0 | 75 | <0.001 | >0.999 | <0.001 | <0.001 |
| Recovery | 7.5 | 8.0 | 69 | 7.6 | 8.0 | 74 | 7.4 | 8.0 | 66 | 0.015 | >0.999 | >0.999 | 0.012 |
| Cardiovascular disease | 7.3 | 8.0 | 60 | 7.1 | 7.0 | 62 | 7.5 | 8.0 | 67 | <0.001 | <0.001 | 0.11 | <0.001 |
| Hospital-acquired infection | 7.2 | 8.0 | 66 | 7.4 | 8.0 | 70 | 7.7 | 8.0 | 75 | <0.001 | >0.999 | <0.001 | <0.001 |
| Fever | 7.1 | 7.0 | 64 | 7.4 | 8.0 | 69 | 7.7 | 8.0 | 74 | <0.001 | <0.001 | <0.001 | <0.001 |
| Chest pain | 7.1 | 7.0 | 58 | 6.6 | 7.0 | 49 | 7.3 | 8.0 | 62 | <0.001 | <0.001 | 0.014 | <0.001 |
| Impact on family | 7.0 | 7.0 | 58 | 7.0 | 7.0 | 59 | 7.1 | 7.0 | 59 | <0.001 | >0.999 | 0.209 | <0.001 |
| Cough | 6.9 | 7.0 | 57 | 7.1 | 7.0 | 61 | 7.3 | 8.0 | 64 | <0.001 | 0.042 | <0.001 | <0.001 |
| Fatigue | 6.7 | 7.0 | 55 | 6.4 | 6.0 | 46 | 6.6 | 7.0 | 48 | <0.001 | <0.001 | >0.999 | <0.001 |
| Life participation | 6.6 | 7.0 | 48 | 6.5 | 6.0 | 48 | 6.5 | 6.0 | 45 | 0.103 |  |  |  |
| Depression | 6.6 | 7.0 | 50 | 6.5 | 7.0 | 49 | 6.5 | 7.0 | 47 | 0.079 |  |  |  |
| Anxiety | 6.5 | 6.0 | 47 | 6.3 | 6.0 | 44 | 6.3 | 6.0 | 41 | 0.041 | 0.034 | 0.093 | >0.999 |
| Gastrointestinal problems | 6.3 | 6.0 | 40 | 6.0 | 6.0 | 36 | 6.3 | 6.0 | 39 | <0.001 | 0.005 | >0.999 | <0.001 |
| Muscle pain | 6.3 | 6.0 | 40 | 6.0 | 6.0 | 35 | 6.2 | 6.0 | 39 | <0.001 | 0.003 | >0.999 | <0.001 |
| Taste and smell | 6.2 | 6.0 | 42 | 6.1 | 6.0 | 38 | 6.4 | 7.0 | 48 | 0.001 | 0.066 | 0.026 | 0.001 |

**^†^**Percentage of respondents rating the outcomes 7 to 9, which indicates critical importance on the Likert scale.

* Kruskal-Wallis test to compare the three groups ** If the Kruskal-Wallis test is significant, pairwise comparisons with the Mann-Whitney test and Bonferroni adjustment.

Note: Total sample size differs for each outcome. Death, Cardiovascular disease, Shortness of breath, Chest pain, Cough n =9,289; Fever, Pneumonia, Hospitalization, Depression, Impact on family n=9,072; Gastrointestinal problems, Fatigue, Life participation, Muscle pain, Respiratory failure n=8,907; Sepsis / septic shock, Taste and smell, Anxiety, Lung function, Recovery n=8,789; Oxygen level in the blood, Viral load or clearance, Lung scarring (fibrosis), Hospital-acquired infection, Organ failure n=8,653.

**eTable 3. Mean and median scores of participants according to language (English, Chinese, Italian, Spanish, Portuguese).** Ordered by English survey mean scores.

|  | **Mean scores** | | | | | **Median scores** | | | | |
| --- | --- | --- | --- | --- | --- | --- | --- | --- | --- | --- |
|  | **English (n=5927)** | **Chinese (n=1480** | **Italian (n=674)** | **Spanish (n=652)** | **Portuguese (n=556)** | **English (n=5927)** | **Chinese (n=1480** | **Italian (n=674)** | **Spanish (n=652)** | **Portuguese (n=556)** |
| Death | 8.5 | 8.1 | 8.0 | 8.1 | 8.3 | 9.0 | 9.0 | 9.0 | 9.0 | 9.0 |
| Respiratory failure | 8.4 | 8.2 | 8.4 | 8.3 | 8.5 | 9.0 | 9.0 | 9.0 | 9.0 | 9.0 |
| Organ failure | 8.1 | 8.0 | 8.0 | 8.2 | 8.2 | 9.0 | 9.0 | 8.0 | 9.0 | 9.0 |
| Pneumonia | 8.0 | 8.2 | 8.5 | 8.2 | 8.4 | 9.0 | 9.0 | 9.0 | 9.0 | 9.0 |
| Sepsis / septic shock | 7.9 | 7.4 | 7.7 | 8.1 | 8.0 | 9.0 | 8.0 | 8.0 | 9.0 | 9.0 |
| Lung function | 7.9 | 7.9 | 8.1 | 7.9 | 8.3 | 8.0 | 9.0 | 9.0 | 8.0 | 9.0 |
| Lung scarring (fibrosis) | 7.8 | 7.9 | 7.9 | 8.1 | 8.0 | 8.0 | 8.0 | 8.0 | 9.0 | 9.0 |
| Shortness of breath | 7.8 | 8.2 | 8.3 | 7.7 | 8.3 | 8.0 | 9.0 | 9.0 | 8.0 | 9.0 |
| Hospitalization | 7.8 | 7.4 | 7.8 | 7.8 | 7.9 | 8.0 | 8.0 | 8.0 | 8.0 | 9.0 |
| Oxygen level in the blood | 7.7 | 7.9 | 8.2 | 7.8 | 8.2 | 8.0 | 8.0 | 9.0 | 8.0 | 9.0 |
| Recovery | 7.6 | 7.3 | 7.7 | 6.9 | 7.9 | 8.0 | 8.0 | 8.0 | 7.0 | 9.0 |
| Viral load or clearance | 7.6 | 7.9 | 8.0 | 7.7 | 8.0 | 8.0 | 9.0 | 8.0 | 8.0 | 9.0 |
| Cardiovascular disease | 7.4 | 6.9 | 7.0 | 7.5 | 7.7 | 8.0 | 7.0 | 7.0 | 8.0 | 8.0 |
| Hospital-acquired infection | 7.3 | 7.7 | 7.9 | 7.6 | 7.9 | 8.0 | 8.0 | 8.0 | 8.0 | 9.0 |
| Fever | 7.3 | 7.9 | 7.8 | 7.4 | 7.9 | 8.0 | 9.0 | 8.0 | 8.0 | 9.0 |
| Cough | 7.0 | 7.8 | 7.0 | 6.7 | 7.8 | 7.0 | 9.0 | 7.0 | 7.0 | 8.0 |
| Impact on family | 7.0 | 7.1 | 7.4 | 7.1 | 7.1 | 7.0 | 7.0 | 8.0 | 7.0 | 7.0 |
| Chest pain | 6.9 | 7.0 | 6.5 | 7.1 | 7.0 | 7.0 | 7.0 | 7.0 | 7.0 | 7.0 |
| Life participation | 6.5 | 6.5 | 6.5 | 6.1 | 6.6 | 7.0 | 6.0 | 6.0 | 6.0 | 7.0 |
| Depression | 6.5 | 6.8 | 6.3 | 6.4 | 6.4 | 7.0 | 7.0 | 7.0 | 6.0 | 6.0 |
| Fatigue | 6.4 | 6.5 | 6.8 | 6.8 | 7.0 | 6.0 | 6.0 | 7.0 | 7.0 | 7.0 |
| Anxiety | 6.3 | 6.5 | 6.2 | 6.3 | 6.4 | 6.0 | 7.0 | 6.0 | 6.0 | 6.0 |
| Gastrointestinal problems | 6.1 | 6.3 | 6.0 | 5.9 | 6.6 | 6.0 | 6.0 | 6.0 | 6.0 | 7.0 |
| Taste and smell | 6.1 | 6.0 | 6.9 | 6.4 | 7.1 | 6.0 | 6.0 | 7.0 | 6.0 | 7.0 |
| Muscle pain | 6.0 | 6.3 | 6.0 | 6.2 | 6.6 | 6.0 | 6.0 | 6.0 | 6.0 | 7.0 |

**eTable 4. Selected quotations for each theme**

| **Theme** | **Comments** |
| --- | --- |
| **Fear of life-threatening, debilitating and permanent consequences** | “This uncertainty causes anxiety and makes breathing harder.” (Shortness of breath, patient, English)  “Definitely. Especially this is what thought to be the main cause of death?” (Respiratory failure, public, English)  “Fact sheet saying not everyone dies. Even though people know this when you have Corona virus you imagine the worse possible scenario, particularly when you are SOB, have chest pain and are bombarded by media.” (Death, patient, English)  “High importance especially as I ended up passing it to my father who ended up in ICU and I did not. Huge psychological, emotional impact” (Impact on family, patient/health professional, English)  “Mental health is important particularly in the confines of isolation wards in hospitals.” (Depression, patient, English)  “You see death coming for you.” (Depression, patient/health professional, English)  “Felt very lonely and depressive about the scenario.” (Depression, patient, English)  “Could lead to PTSD, also future organ damage.” (Sepsis, public, English)  “Are there any permanent long-term consequences on the respiratory system?” (Lung function, patient/health professional, English)  “As it takes a while for lungs to heal after being damaged by COVID-19.” (Lung function, patient, English)  “Feeling of imminent death, fear, psychological distress and physical pain.” (Shortness of breath, health professional, Italian)  “I think that is like feeling dying.” (Shortness of breath, patient, Italian)  “Desperation and fear of never seeing family and friends again.” (Depression, health professional, Italian)  “It could be hard to resume their own duties and roles.” (life participation, health professional, Italian)  “[Respiratory failure causes] suffering and fright for the worsening of their own condition.” (Respiratory failure, health professional, Italian)  “Like I said before, if you breathe badly, you live badly.” (Lung scarring, patient, Italian)  “It is exasperating to see how they cannot breathe.” (Shortness of breath, heath professional, Spanish)  “It is important because you realize the seriousness of the situation.” (Respiratory failure, public, Spanish)  “I don't know if I had it, but when the shortness of breath came I thought I was going to die, or when my throat got stuck.” (Death, public, Portuguese)  “Economic and family impact.” (Depression, health professional, Portuguese)  “Little by little getting better, but I thought I would die.” (Lung function, public, Portuguese)  “[It is] present due to the fear of dying and not to have a specific remedy.” (Anxiety, health professional, Portuguese)  “Mental health can lead to extreme situations.” (Depression, public, Portuguese) |
| **Addressing knowledge gaps** | “Two of my patients have died. I need to understand the pathophysiology of this disease better.” (Death, health professional, English)  “Need to know if someone died with COVID-19 or of COVID-19.” (Death, health professional, English)  “But only if it can be directly attributed to COVID-19.” (Respiratory failure, public, English)  “To understand the influence of previous disease.” (Death, public, Italian)  “The symptoms are mainly respiratory and circulatory symptoms, which can be refined” (health professional, Chinese)  “Some outcome ratings are really difficult for the unknown disease” (health professional, Chinese)  “The family is scared because they think they are also carriers of the virus and the same will happen to them.” (Hospitalization, health professional, Spanish)  “They are not aware of the severity.” (Shortness of breath, health professional, Spanish)  “The feeling of helplessness and insecurity in the absence of answers from science, if scientists do not know and change their messages; the future is uncertain.” (Health professional, Spanish)  “It is important to know the impact of COVID-19 in people with this condition, in terms of the probability of recovery / death.” (Cardiovascular disease, public, Portuguese)  “As the main symptom; must be accompanied by information on asymptomatic population.” (Shortness of breath, public, Portuguese)  “You will be able to inform future studies on mortality, which will be extremely necessary, not only now in the acute phase, but certainly at a later stage and for [the following] years (Impact on family, health professional Portuguese) |
| **Enabling preparedness and planning** | “Important as this would inform my decision to continue very invasive treatment or not.” (Death, public, English)  “Helps identify when you can leave isolation.” (Recovery, patient, English)  “Very important because it informs all measures that should be taken (lockdown, production of ventilators etc.)” (Respiratory failure, public, English)  “To help other areas predict how much equipment they might need.” (Respiratory failure, health professional, English)  “Death rates within the population help to plan accordingly.” (Death, health professional, English)  “Essential for better planning.” (Hospitalization, public, English)  “Helps inform medical decision-making and planning” (Organ failure, health professional, English)  “Helping with decision-making for patient and loved ones to use ventilation.” (Respiratory failure, health professional, English)  “[It means] add extra burden to an already serious situation ...” (Hospital-acquired infection, patient, Italian)  “It’s critical if we are able to diagnose COVID-19 infection at an early stage so that we could treat it in time” (Patient/health professional, Chinese)  “Shortness of breath, cough, chest pain are directly related to COVID-19, and help a lot in diagnosing quickly” (Clinician, Chinese)  “It is acting late and wrong, without enough protective equipment and with few diagnostic tests.” (Public, Spanish)  “It took a long time to get the ambulance to the hospital” (Patient, Spanish)  “To assess the capacity of hospital facilities in preventing the spread of infections.” (Hospital-acquired infections, public, Portuguese)  “I assume that an early approach would reduce the need for an ICU” (Cough, Chest pain and Cough, health professional, Portuguese) |
| **Tolerable or infrequent outcomes** | “Not critical, compared to others unless it is longer term.” (Taste and smell, patient/family member, English)  “Cough is a common symptom, and is already known as the primary infection method of person-to-person transmission.” (Cough, health professional, English)  “Treatable to a degree.” (Muscle pain, health professional, English)  “Was temporary.” (Fever, patient/family member, English)  “We need to be able to assist patients with this symptom, but I'm not sure of the value of it as a marker for covid-19 as it is very non-specific.” (Anxiety, health professional, English)  “I had minimal symptoms with this so I am not too fussed about it.” (Gastrointestinal, patient/health professional, English)  “Not apparent in every case.” (Fever and CVD, family member, English)  “It has an impact but compared with shortness of breath you can cope with that.” (Life participation, patient, Italian)  “It is annoying, but [it is] less harmful than the other problems already listed.” (Muscle pain, patient, Italian)  “Unfortunately, there are certain parameters of COVID that in our population are not occurring as frequently such as fever.” (Fever, heath professional, Spanish) |

**eFigure 1. Proportion % 7-9 scores (critically important) of patients/family members, health professionals and public.** Ordered by patient/family scores. Score of 7 to 9 indicates critical importance on the Likert scale.

HAI: Hospital-acquired infection. Total sample size differs for each outcome. Death, Cardiovascular disease, Shortness of breath, Chest pain, Cough n =9,289; Fever, Pneumonia, Hospitalization, Depression, Impact on family n=9,072; Gastrointestinal problems, Fatigue, Life participation, Muscle pain, Respiratory failure n=8,907; Sepsis / septic shock, Taste and smell, Anxiety, Lung function, Recovery n=8,789; Oxygen level in the blood, Viral load or clearance, Lung scarring (fibrosis), Hospital-acquired infection, Organ failure n=8,653

**eFigure 2. Relative importance scores of participants according to language (English, Chinese, Italian, Spanish, French) based on the Best-Worst Scale.**


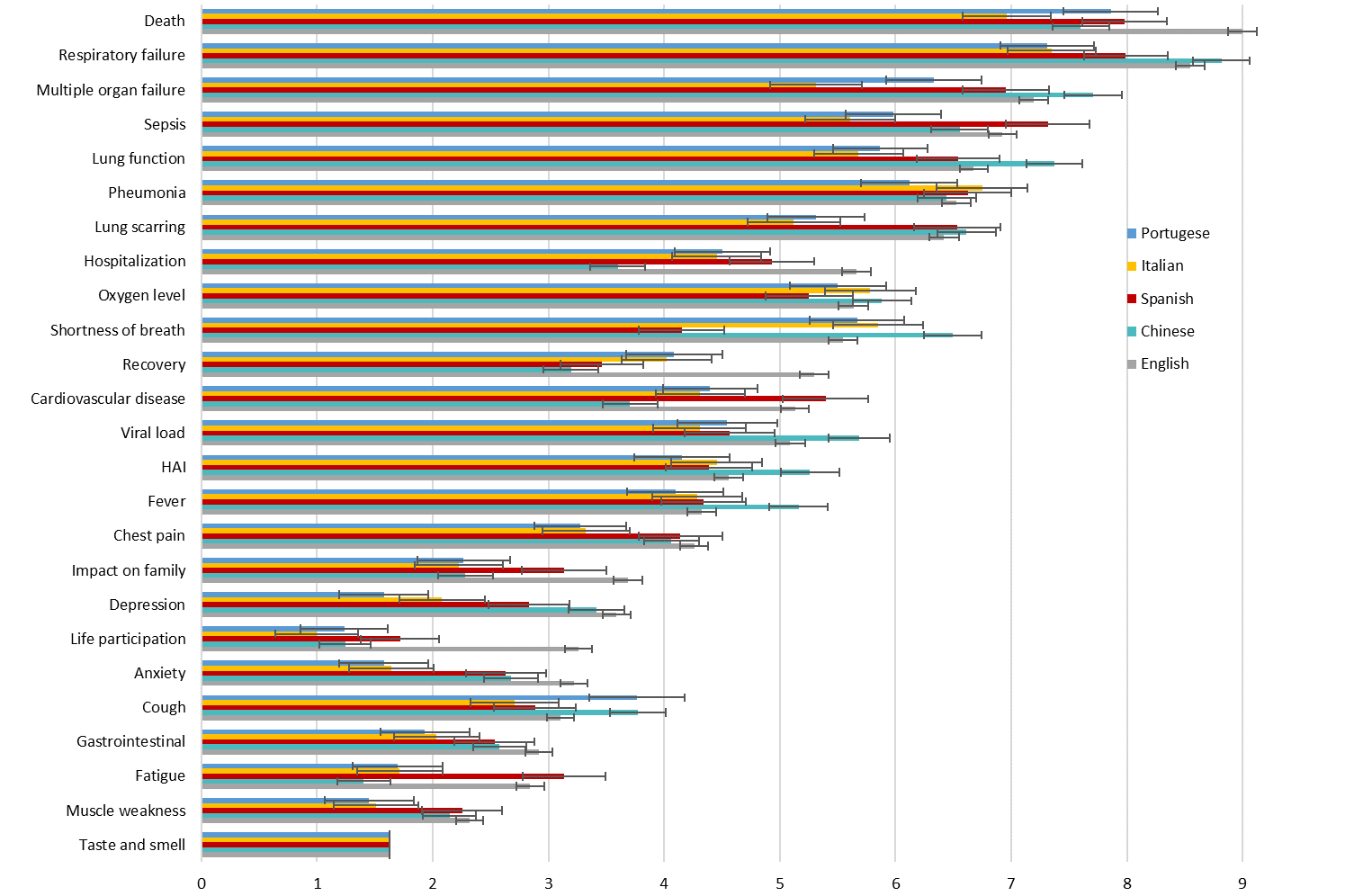

Supplement: Supplementary file 1 [file ccm-48-01612-s001.docx]
